# Fiber Bragg Grating Sensor for Fault Detection in Radial and Network Transmission Lines

**DOI:** 10.3390/s101009407

**Published:** 2010-10-20

**Authors:** Amin A. Moghadas, Mehdi Shadaram

**Affiliations:** Department of Electrical & Computer Engineering, University of Texas at San Antonio, TX 78249, USA; E-Mail: mehdi.shadaram@utsa.edu

**Keywords:** current measurement, current transformers, optical fiber, magnetostrictive devices, power system protection

## Abstract

In this paper, a fiber optic based sensor capable of fault detection in both radial and network overhead transmission power line systems is investigated. Bragg wavelength shift is used to measure the fault current and detect fault in power systems. Magnetic fields generated by currents in the overhead transmission lines cause a strain in magnetostrictive material which is then detected by Fiber Bragg Grating (FBG). The Fiber Bragg interrogator senses the reflected FBG signals, and the Bragg wavelength shift is calculated and the signals are processed. A broadband light source in the control room scans the shift in the reflected signal. Any surge in the magnetic field relates to an increased fault current at a certain location. Also, fault location can be precisely defined with an artificial neural network (ANN) algorithm. This algorithm can be easily coordinated with other protective devices. It is shown that the faults in the overhead transmission line cause a detectable wavelength shift on the reflected signal of FBG and can be used to detect and classify different kind of faults. The proposed method has been extensively tested by simulation and results confirm that the proposed scheme is able to detect different kinds of fault in both radial and network system.

## 1. Introduction

Current transformers (CTs) and potential transformers (PTs) are widely used in monitoring power systems by sending fault current/voltage information to relay and control rooms at substations. If current/voltage in a particular line is out of a pre-set range, relays will send a trip signal to breakers to trip. If a primary relay fails to operate and clear a fault, based on pre-set rules, a back up breaker will clear the fault. Conventional CTs are iron based which subject to saturation and hysteresis. Most relays make decisions and send trip signals based on the root mean square (rms) value of fault current detected by the CT. Saturation in the CTs cause the rms value of fault current sensed by the CT to be much smaller than the actual value and it can prevent relays from tripping and eventually cause instability in the system.

Optical current transformers (OCT) are becoming more popular in power systems. An OCT can offer a better transient response, better accuracy, and wider bandwidth in comparison to traditional CTs due to the OCTs’ lack of iron core [1]. OCTs are light, small, less expensive, and immune to electromagnetic interference (EMI) [2]. Already, a number of Faraday effect current sensors have been investigated and successfully implemented [3,4].

Although OCTs can measure high current without saturation, lack of proper fault detection and classification algorithm prevents them to apply in power systems. Previous researchers have discussed fault effect in the FBG wavelength shift [5–7]. Authors here, for the first time, use wavelet transforms and ANN algorithm to detect and classify the faults based on FBG wavelength shift signals. This paper, proposes an FBG based OCT which can replace conventional CTS. A unique ANN algorithm is used to first detect the fault based on FBG wavelength shift and then classify it. The proposed OCT does not need any CT or PT for biasing and the proposed ANN algorithm works in both radial and network systems.

## 2. Magnetostrictive Materials

Magnetostrictive materials are the part of ferromagnetic materials that transform from one shape to another in the presence of the magnetic field. Magnetic field causes internal strain in the magnetostrictive material with consequences of expansion of the material in the magnetic field direction. In magnetostrictive materials, magnetic field strength is proportional to the square of applied strain until eventually the magnetic saturation achieved. Since the basis of expansion is molecular, the magnetostrictive materials are very sensitive to strain and have a very fast response [8,9]. Also, due to the change in the crystal structure of the material, measurement is repeatable with in milliseconds. Among these materials, Terfenol-D, *Tb*_0.3_*Dy*_0.7_*Fe*_1.95_, an alloy of Terbium, Dysprosium and Iron, has the highest strain in magnetic field. At room temperature, Terfenol-D can produce about 1,000 ppm which is large enough to apply to FBG strain sensor. Previously, Sun and Zheng [10] have shown that the highest sensitivity in the Terfenol-D in the magnetic field up to 20 kA/m can be achieved with 6.9 Mpa prestress. Due to the nature of all giant magnetostrictive materials, applying prestress can cause a better sensitivity. However, their response is roughly proportional to the strength of the magnetic field. Terfenol-D can be polarized by using a DC biasing field [8,9]. Performance of the Terfenol-D depends on the prestress and the DC bias magnetic field.

**Figure 1.**
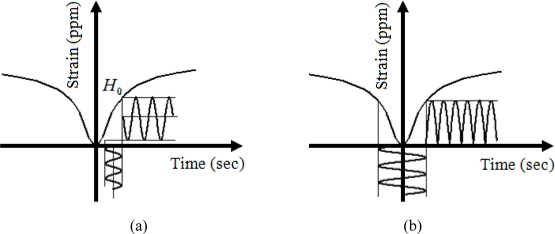
Biasing a Terfenol-D **(a)** with the DC biasing; **(b)** without the DC biasing.

**Figure 2.**
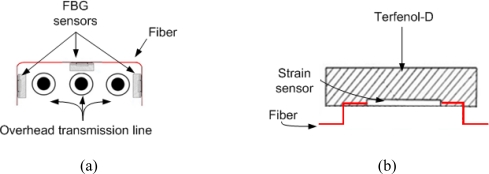
**(a)** Sensor placement in the overhead transmission line. **(b)** Sensor detail design including Terfenol-D, fiber optic cable, and strain sensor.

Since AC magnetic field is measured in this experiment, the DC biasing is necessary to shift the AC wavelength and prevent changing the polarity of the output while the input is changing. DC biasing can be achieved with serial or parallel permanent magnet, or the DC biasing coil. In this experiment, the DC biasing method is used due to its simplicity to change the DC magnetic field. In general, biasing point of the magnetostrictive material is defined based on applied prestress and DC biasing point. Figure 1(a,b) show Terfenol-D with and without DC biasing field respectively. As shown in Figure 1(a), the DC biasing field, *H*_0_, is chosen such that the slope of the curve is in its maximum point. Behavior of magnetostrictive materials is a nonlinear relation which is already described in detail [10,11]. The structural design of FBG sensor using Terfenol-D has been shown in the Figure 2(b). In this experiment, DC solenoid with 2,600 amp-turn produces the DC biasing field and causes wavelength shift in FBG strain sensor attached to Terfenol-D material. The Hysteresis and eddy losses are present in the giant magnetostrictive materials [12–14] and they are considered in the sensor model.

## 3. FBG Sensors

FBG sensors can work as arrays for real time measurement of temperature, strain, and pressure in the systems. Optical fiber sensors have numerous advantages such as electrically passive operation, EMI immunity, high sensitivity, and multiplexing capabilities which make them a perfect candidate to use in power systems. FBG sensors are commonly used for strain and temperature measurement and they can measure strain up to ±5,000 *μ*ε and temperature ranges from −40 °C to +120 °C Strain causes change in the grating pitch and the fiber index of the sensor. The sensed strain in FBG sensor is then coded directly into the wavelength and can be detected as wavelength shift. FBGs reflect a narrowband of light and transmit all other wavelengths. In other words, FBG is an optical fiber that works as a filter for a particular wavelength. The principal of a FBG based sensor is to detect the reflected Bragg wavelength shift due to changes in temperature, strain, or pressure. The Bragg wavelength is defined as follows [15].

(1) $$\lambda_{B}=2n_{\mathrm{eff}}\Lambda$$

where, Λ is the grating pitch and *n_eff_* is the effective index of the fiber core. Bandwidth of the Bragg reflected signal depends mainly on grating length and it is typically around 0.05 to 3 nm.

OCT used in this experiment consists of FBG strain and temperature sensors and a giant magnetostrictive material bounded together with epoxy. As described earlier, biasing point of the sensor can be adjusted by mechanical prestress on magnetostrictive material and the amp-turn of the DC biasing solenoid. One of the main advantages of the OCT sensor, which can substantially reduce the cost of implementing power system, is that the OCT sensor does not require any CTs and PTs for biasing to detect the fault in the system. Terfenol-D (*Tb_x_Dy*_1−_*_x_Fe_y_*) was chosen as the magnetostrictive material due to its fast response (<0.1 ms) [16,17] and capability of producing a substantial amount of strain in the magnetic field. A broad band source was used to illuminate the FBG through a single mode fiber optic and optical coupler. The principle of FBG is well known [15] and will not be explained in detail here. A small change in strain and temperature both cause wavelength shift on the reflected FBG wavelength by following equations:

(2) $$\frac{\Delta \lambda }{\lambda_{0}}=kɛ+\alpha_{\mathrm{nTemp}}\Delta T.$$

(3) $$\alpha_{\mathrm{nTemp}}=\frac{\frac{\delta n}{n}}{\delta T}$$

where, Δ*λ* is the wavelength shift, *λ*_0_ is base wavelength in nominal temperature and strain, Δ*T* is the temperature change, k is the gage factor which is normally 0.78, *α_nTemp_* is the change in index of refraction with respect to temperature, and *ε* is strain. In this paper, the following parameters were measured through experiment: *λ*_0_=1528 nm, Δ*λ*=1.17*pm*/(*μstrain*), and wavelength shift is Δ*λ=*13.48pm/°*C*. Also, separate FBG temperature sensor with 27.1 mm probe, ±1.7*pm*/°*C* sensitivity and 0.3 s response time is used to measure temperature and compensate temperate effect. The system is calibrated for ambient temperature of 25° degree.

## 4. Development of the Concept

In each phase of the overhead transmission line one sensor is installed and all the sensors should be placed as close as possible to its own conductor as shown in the Figure 2(a). They should be placed as far as possible from the remaining conductors. Detailed design of sensors and their placement have been shown in the Figure 2(b). Since magnetic field is inversely proportional to the distance squared, the effect of other phases in this sensor is so small they can be easily ignored in the simulations. AC current measurement with FBG has been extensively discussed earlier [18–23]. If overhead transmission line is assumed straight infinite conductor, its magnetic field can be expressed as:

(4) $$H=\frac{I}{2\pi d}$$

where, d is the distance from the center of Terfenol-D to the conductor. In our study, two FBG current sensors installed at both ends of the transmission line sense strain in both ends of transmission line. The transmission line is an assumed bundle with geometer mean diameter (GMD) of 11.07 m and geometric mean ratio (GMR) of 0.127 m with length of 150 km aluminum conductor, steel reinforce (ACSR), 60 Hz, and 0.55 per unit impedance. Base values for the system are: 100 MVA and 138 kV.

**Figure 3.**
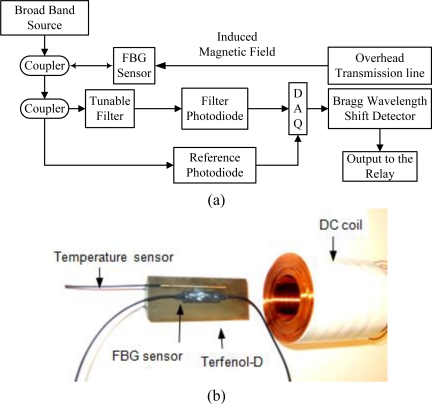
**(a)** Block diagram of the system; **(b)** Experimental setup. (DC coil, Terfenol-D rod, strain sensor, and temperature sensor).

The block diagram of the system and experimental setup are shown in Figure 3(a,b). *V_reference_* and *V_filter_* are the output of the reference photodiode and filter photodiode, respectively. The reflected light (*V_reference_*) is sent through a separate path to the reference photodiode. Previously, Melle and Liu [24] have shown that the ratio *V_filter_* */V_reference_* is proportional to the fiber Bragg wavelength shift:

(5) $$\frac{V_{\mathrm{filter}}}{V_{\mathrm{reference}}}=K(\lambda_{B}-\lambda_{0}+\frac{\Delta \lambda }{\sqrt{\pi }})\;.$$

where, constant K is the slope of the edge or tunable filter, *λ*_0_ is the wavelength at which the edge filter has zero transmittance, *λ_B_* is the peak wavelength of the grating, and Δ*λ* is the spectral width of the Bragg reflected spectrum. This method has been chosen to measure the Bragg wavelength shift because of its simplicity to install, less complexity and fast response. In this experiment, a DC coil made of copper AWG 24 wire with 2,600 amp-turn is used with excitation current of 0.55 A and resistance of 18.8 Ω.

## 5. Simulation Result

### 5.1. Radial System

A typical high voltage power system using electrical transient analyzer program (ETAP) is simulated with the assumption of delta-wye connection for all transformers and delta connection for all loads in the system. Horizontal configuration is used and the sensors are placed 3 cm away from each phase.

**Figure 4.**
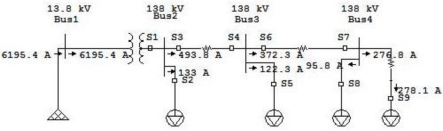
One line diagram of radial system with sensor location.

With changing the DC biasing point of the sensor, this method can be applied to different pole structures, different cable sizes and cable configurations. A typical radial system with 4 buses is shown in Figure 4. In this simulation all equipment is effectively grounded and three sensors are considered for each transmission line.

All the sensors are installed close to the protection relay and are shown with word S followed with the sensor number. OCTs are shown by small squares in the system which is installed in all three phases and they continuously monitor the magnitude and the phases of the system.

Increase in the current due to fault in part of the system will cause an increase in the magnetic field around the cable. When a short circuit happens in the overhead transmission line, the Bragg wavelength of OCT sensed by downstream sensors is shifted due to drastic current change in the system. All different kinds of fault between sensor *S*_3_ and *S*_4_ including single line to ground (SLG), double line (DL), and three line to ground have been simulated and the sensors response have been shown. The High voltage power system investigated in the paper is assumed to operate at the normal condition and the systems are balanced with positive sequence. Since the power system is assumed to be balanced and power system analyzing software such as ETAP assume the system in normal operation stays balanced, all sensors sense the same wavelength shift for all phases.

**Figure 5.**
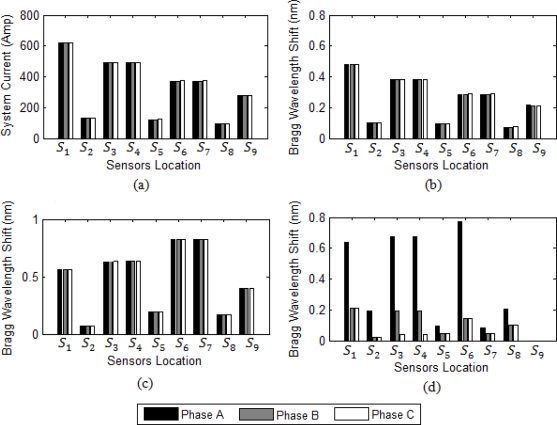
Fault response at bus 4. **(a)** System current in the normal condition. **(b)** Bragg wavelength magnitude for each sensor in normal operation of the system. **(c)** Sensor Bragg wavelength magnitude in a three phase to the ground fault at bus 4. **(d)** Sensor Bragg wavelength magnitude in a SLG fault at bus 4.

After short circuit calculation for a power system with ETAP, then all signals are sent to Matlab for further processing. Normal operation of the system and sensed current are shown in Figure 5(a).

Multiple sensors can use one fiber optic strand due to the straightforward ability of multiplexing several optical current sensors on one optical fiber. Figure 5(b) shows Bragg wavelength shift in normal operation of the system. In three phase fault and three phase to ground fault, system current stays balanced and it is the same for each phase. As a result, the wavelength shifts detected by all the sensors in aforementioned faults stay the same as shown in Figure 5(b). However, in one line to ground fault, two line fault, and two line to ground fault current, the system is unbalanced causing different wavelength shift for each phase. Figure 5(c) shows when a three phase to ground fault happens in the transmission line at bus number 4. Since the system is radial, all upstream feeders and buses should sense the high short circuit current.

The highest surges at sensors *S_6_* and *S_7_* hint the closest sensor to fault location are both *S_6_* and *S_7_*. Figure 5(d) shows the FBG wavelength shift when the single to ground fault (SLG) happens between phase A and ground. The highest surge of wavelength shift is in sensor *S_6_* and it therefore shows that the fault is the closest to this sensor. Other upstream sensors and their corresponding breakers can work as back up in the short circuit fault of the system.

**Figure 6.**
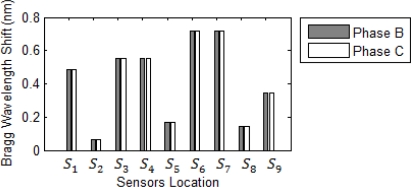
Bragg wavelength shift in a DL fault.

Figure 6 shows DL fault between two phases B and C in the same transmission line at bus 4. Like all earlier cases, the highest current surge in the system causes the highest wavelength shift resulting at the closest sensor to the fault. The highest surge in this system is sensed by *S_6_* and *S_7_* and the current in both phases is increased which means DL fault are happened in the system.

### 5.2. Network System

**Figure 7.**
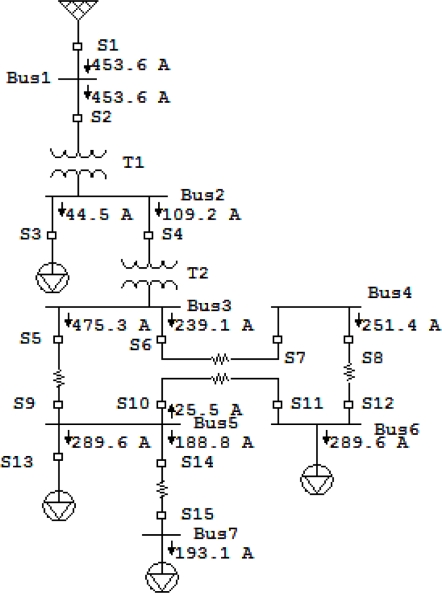
One line Diagram of the system with sensors location and their current in normal operation of the system.

In Figure 7 the one line diagram of simple 60 Hz network system with 7 buses has been shown; Both 138/13.8 kV transformer in the system considered having unique leakage reactance of 0.06 pu. Transmission line is assumed bundle with GMD = 11.07 m and GMR = 0.127 m with length of 150 km aluminum conductor, steel reinforce (ACSR), 60 Hz with impedance of 0.55 pu. Base values for this system are the same as the radial system: 100 MVA and 138 kV.

**Figure 8.**
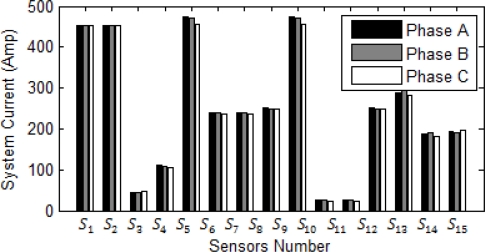
Load flow of system in the normal operation.

**Figure 9.**
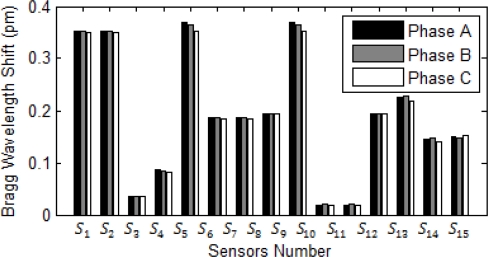
Bragg wavelength magnitude in the normal operation of the system.

**Figure 10.**
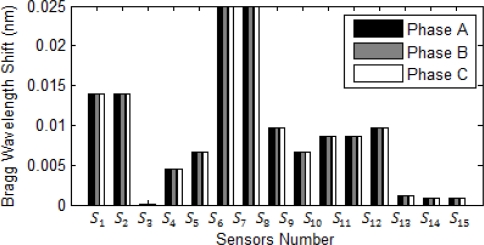
Bragg wavelength magnitude in the three phase fault at bus 4.

Newton-Raphson load flow method with precision of 0.001 has been used. One line diagram and the load flow of the system in normal operation and the Bragg wavelength shift of corresponding sensors have been shown in Figure 7, Figure 8 and Figure 9, respectively. In all faults, the magnitudes of Bragg wavelength shift detected by the sensors are increased in comparison to either steady state or normal operation of the system. Figure 10 indicates three phase fault at bus 4 near sensor *S_7_*.

**Figure 11.**
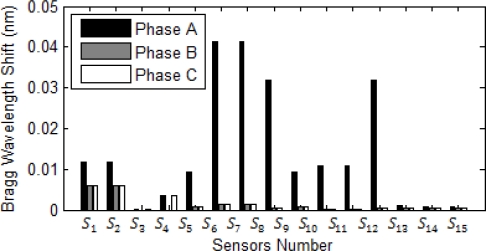
Bragg wavelength magnitude in the SLG fault at bus 4.

**Figure 12.**
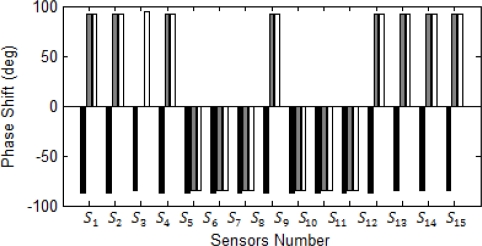
Sensor phases in the SLG fault at bus 4.

Figure 10 shows the magnitude each sensor exceeds from its normal operation due to increase in the current. All current in the approximate of this bus sense huge Bragg wavelength shift, except *S_3_* which is connected to the bus feeding motors and in the short circuit motors always act as generators. Figure 11 and Figure 12 show magnitude and phase of sensor at single line to ground fault (SLG) at bus 4 (phase A fault to the ground at bus 4.) As shown, all sensors connected to phase A of the system see a wavelength shift while the other two phases do not sense major wavelength shift. Wavelength shifts simply reveal that the concerning faulted phase is phase A of system.

Figure 11 and Figure 12 reveal that the highest surges happen at sensor *S_6_* and *S_7_* and the second highest surge is at sensor *S_8_* and *S_12_*. Since bus 6 is part of the network system, short circuit current can flow to the bus 6 from source to the ground through sensors *S_6_*, *S_7_*, *S_8_* and *S_12_*. Thus, the fault location limits to these sensors and it should be somewhere between of all aforementioned sensors.

**Figure 13.**
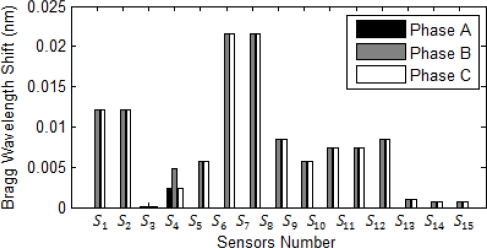
Bragg wavelength magnitude in a DL fault at bus 4.

**Figure 14.**
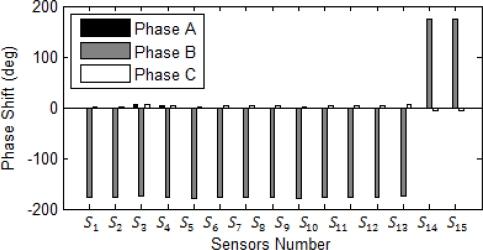
Sensor phases in a DL fault at bus 4.

Phase reading should be used to detect DL fault in the network system. Figure 13 and Figure 14 illustrate magnitude and phase of sensor response under DL fault at bus 4, respectively. As expected, the highest surges detected by the sensor are in the sensors *S_6_* and *S_7_*. The result hints that the fault should be at the bus close to these sensors. At DL fault, both phases at the same time can sense the same magnitude of surge for both phases A and B. Similar to the previous case, the second highest shifts are at the sensors *S_8_* and *S_12_*. Based on the topology of the system, the faulted location can be easily recognized and limited to sensors *S_6_*, *S_7_*, *S_8_*, and *S_12_*; however, the sensor *S_1_* and *S_2_* sensed the higher shift in comparison to the sensor *S_8_* and *S_12_*, they should be ignored since they are not close to the reference sensors *S_6_*, *S_7_* nor did their phase reading reveal fault location close to these sensors. All sensors around the faulted bus confirm that the current direction is toward the faulted bus. In this research high harmonic current sensors mal-function and backup protection in the ANN network were not considered which limit the research. Future research should address the harmonics effect at the sensor response, sensor mal-function, and sensor noise based on different pole structure and configuration.

### 5.3. Fault detection and Classification Algorithm

All fault detection algorithms have two main tasks including detection and classification. Previously, numerous algorithms for transmission line fault detection using artificial neural network (ANN), wavelet transform (WT) [25], ANN and fuzzy logic [26], ANN and WT [27] have been discussed. The fault detection method discussed here, uses FBG wavelength shift data as an input to an ANN algorithm.

**Figure 15.**
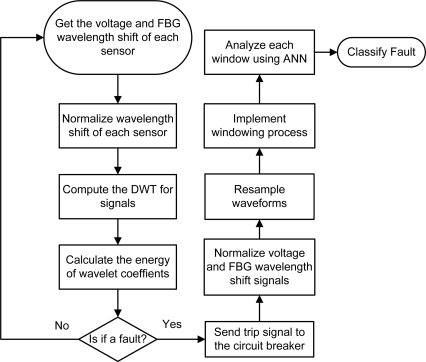
Flowchart of the proposed fault detection method.

In the classification module the voltage and the Bragg wavelength shift samples are recorded. Then the samples are normalized by the largest value in the record and the waveforms are resampled for 1,200 Hz (20 samples/cycle for a fundamental frequency of 60 Hz). A windowing of the samples form a set of inputs to the ANN (phase and zero sequence components). A multilayer perceptron (MLP) analyzes the input samples one by one and at the end the final fault classification is defined by the most identified fault type. Both voltage and the FBG wavelength shift samples are normalized to their peak values at the first cycle in the record, corresponding to the steady state mode of the power system. In both high and extra high voltage transmission systems, fault in the system can cause a voltage sag in the system and also in some cases some voltage sags induce a large high frequency on both voltage and current signals. In addition, this paper has shown that the short circuit in the system can cause detectable FBG wavelength shift in the sensor. The fault detection rules are concluded by analysis of the FBG wavelength shift in time domain and then in the first decomposition level of discrete wavelet transform (DWT) which contains the highest frequency component. Previously, a successful method includes a combination of wavelet and ANN for classification, and detection of faults have been implemented and tested in the real power network [27]. A similar algorithm is proposed here as shown in Figure 15; however, wavelength shift has been used in lieu of current. The fault detection algorithm can be summarized as follows:

1. If Δ*λ_pos_* ≈ Δ*λ_pre_*, it is not a fault.
2. If *E*_max_ < E^*^, it is not a fault.
3. If *E*_max_ ≥ E^*^and Δ*λ_pos_* ≤ 0.14Δ*λ_pre_*, it is not a fault.
4. If *E*_max_ ≥ E^*^and|Δ*λ_pos_* − Δ*λ_pre_*| < 0.14 max{Δ*λ_pos_*, Δ*λ_pre_*} it is not a fault.
5. If *E*_max_ ≥ E^*^, and none of aforementioned rules have not met, it is a fault.

Here, the *E*_max_ is the maximum energy of the FBG wavelength shift wavelet coefficients, E^*^ is the threshold energy defined by the analysis of the wavelet coefficient energy in different system operations such as various kinds of fault, and maintenance in part of the system; Δ*λ_pos_* and Δ*λ_pre_* are the FBG wavelength shift values on the first and last recording wavelength shift cycles.

To calculate the energy of the wavelength shift, a moving window goes through the wavelength shift wavelet coefficients shifting one coefficient each time:

(6) $$E_{i}=\sum_{n=1}^{n_{T}}{\left[d_{i}(n)\right]}^{2}$$

Where, *d_i_*(*n*) is the *n*th wavelet coefficient within the *i*th window and the window length is *n_T_* which is defined by *n_T_*= *n_s_*/2 and *n_s_* is the number of samples within one cycle of fundamental frequency of 60 Hz. For classification purposes like detection module, both voltage and the FBG wavelength shift samples are normalized to their peak value at the first cycle in the record. MLP network should be trained before implementing the proposed method. To classify all faults correctly, the learning data base should contain all varieties and different kinds of fault scenarios to improve ANN capability [28,29]. Output of ANN shows which type of fault is related to the input samples and in this study binary coding is used for the ANN’s outputs. In the case of presence and absence of fault the ANN’s output will be 1 and 0, respectively. All kinds of faults in binary format have been shown in Table 1.

Daubechies 4(db4) [30] mother wavelet is used in the simulation and numerous real records obtained from different transmission lines have been used to establish the detection rule threshold E^*^ and the value is chosen 0.11. To perform the learning process and build the learning data base, faults are simulated in the 138 kV line, 150 km long. Different topologies have been investigated and the highest and most accurate result has been achieved by using a topology with 40 neurons in the hidden layer. Results of the training, validation, and testing have been shown in Table 2. In total, 420 faults in bus 4 of radial and then network overhead transmission line were simulated and 99% success were achieved in detection and classification of fault in both feeder type as shown in Table 3 and Table 4, respectively.

## 4. Conclusions

A novel protection method in the overhead power transmission line dependent in FBG optical current sensor that uses magnetostrictive material is investigated. These FBG current sensors can monitor the whole power system simultaneously and they offer better accuracy, and wider bandwidth in comparison to conventional CTs. Both radial and network power systems are designed with ETAP, and FBG wavelength shifts are processed with MATLAB software. Simulation and signal processing results both confirm the effectiveness of the proposed method. Fault detection rules have been defined based on ANN algorithm to detect the faulted bus and the rules almost always were successful. However, misclassification can occur in the cases that the incident angle, or fault resistance are different than what is used during the ANN’s learning process.

Both the DC biasing point and the prestress should be defined based on the normal current operation of the investigated power system. Higher performance can be achieved by precisely defining prestress in magnetostrictive material, accurately adjusting the DC biasing point of the sensor, and evaluate all possible disturbances in the system. Sensors can detect all kinds of fault in both radial and network overhead transmission line irrespective of the fault type, fault location, and fault resistance up to 100 Ω. The main advantages of the FBG current sensors include their ease of installation, immunity to EMI, reduced insulation requirement, smaller size, and they are less expensive in comparison to conventional CTs. In this research high harmonic current, sensor mal-function, complex feeder network and backup protection in the ANN network were not considered which limit the research. Future research should address the harmonics effect at the sensor response, sensor mal-function, and sensor noise based on different pole structure and configuration.

## Figures and Tables

**Table 1.** Ann’s binary output of the system.

**Fault type**	**Phase A (output 1)**	**Phase B (output 2)**	**Phase C (output 3)**	**Ground (output 4)**
AG	1	0	0	1
BG	0	1	0	1
CG	0	0	1	1

AB	1	1	0	0
AC	1	0	1	0
BC	0	1	1	0

ABG	1	1	0	1
ACG	1	0	1	1
BCG	0	1	1	1

ABC	1	1	1	0
No Fault	0	0	0	0

**Table 2.** Simulation data set.

**Variables**	**Training**	**Validation**	**Test**
Fault location (km)	10-30-50-70-90 100-120-140	60–110	80–130
Fault type	AG-BG-CG-AB-BC-AC-ABG-BCG-ACG-ABC		
Fault resistance (Ω)	Phase-Phase: 0.5 and 5 Phase-Ground: 10, 50 and 100		

**Table 3.** Transmission line fault detection results in the radial system.

**Real system change**	**Expected change in system**	**Number of iterations**	**Number of Success**

Voltage sag	No fault	45	45
Line de-energization	No fault	70	70
Normal operation	No fault	25	25
AG fault	AG fault	70	70

AB fault	AB fault	70	68
ABG fault	ABG fault	70	68
ABC fault	ABC fault	70	70

		**420**	**416**

**Table 4.** Transmission line fault detection results in the network system.

Real system change	Expected change in system	Number of iterations	Number of Success

Voltage sag	No fault	45	45
Line de-energization	No fault	70	70
Normal operation	No fault	25	25
AG fault	AG fault	70	69
AB fault	AB fault	70	67
ABG fault	ABG fault	70	67
ABC fault	ABC fault	70	70

		**420**	**413**
